# The specificity triad: notions of disease and therapeutic specificity in biomedical reasoning

**DOI:** 10.1186/1747-5341-9-14

**Published:** 2014-10-18

**Authors:** Shai Mulinari

**Affiliations:** 1 Department of Sociology, Faculty of Social Sciences, Lund University, Box 114, Lund, 221 00, Sweden; 2 Department of Clinical Sciences, Unit of Social Epidemiology, Faculty of Medicine, Lund University, Malmö, Sweden

**Keywords:** Specificity, Style of thought, Biomedicine, Fleck, Regenerative medicine, Developmental biology, Psychiatry, Neuroscience

## Abstract

Biomedicine is typically defined as the branch of medicine that is based on the principles of biology and biochemistry. A central tenet for biomedicine is the notion of disease and therapeutic specificity, i.e. the idea of tailored treatments for discrete disorders underpinned by specific pathologies. The present paper is concerned with how notions of disease and therapeutic specificity guide biomedical reasoning. To that end, the author proposes a model – the specificity triad – that draws on late philosopher and physician Ludwik Fleck’s concept of “style of thought” to offer a frame for investigating the intricate process through which links between disorders, mechanisms, and therapeutics are established by biomedicine. Next by applying the specificity triad model to scrutinize research efforts in two discrete areas of medicine—psychiatry and regenerative medicine—this paper seeks to stimulate pertinent discussions in and about biomedicine. These include discussions on the ambiguous epistemic status of psychiatry within contemporary biomedicine, as well as the relationship between developmental biology — historically relatively disjointed from biomedical enterprise — and the burgeoning field of regenerative medicine.

## Introduction

Biomedicine is typically defined as the branch of medicine based on the principles of biology and biochemistry. Although the word biomedicine did not gain real currency until the second half of the twentieth century, historians have tracked the roots of biomedicine to the nineteenth century when biology and biochemistry became important resources in clinical medicine [1]. Several commentators from both inside and outside medicine have noted how the ascendance of biomedicine brought with it an increased emphasis on disease and therapeutic specificity, i.e. the idea of tailored treatments for discrete disorders underpinned by specific pathologies [2-4]. Thus a chief goal of biomedicine has been to develop “magic bullet” therapies, a termed coined at the turn of the twentieth century by Paul Ehrlich, the discoverer of tetanus antitoxin and arsenic treatment of syphilis, to describe drugs that would attack the pathogenic invader without damaging its human host [5]. However, to date, there have been few attempts to connect this observation to theories from the philosophy of science on the nature of scientific reasoning.

Against this background, the primary aim of this paper is to outline an analytic frame that draws on Ludwik Fleck’s philosophy of science [6] and that may help address how, more precisely, a commitment to disease and therapeutic specificity structures biomedical reasoning. In particular, the aim is to offer a frame for investigating the intricate process through which *links* between disorders, mechanisms, and therapeutics are being worked out in biomedicine. A secondary aim is to employ this frame to disentangle some ongoing discussions in and about biomedicine. These include pertinent debates about the ambiguous epistemic status of psychiatry within contemporary biomedicine, as well as the relationship between developmental biology — historically relatively disjointed from biomedical enterprise — and the burgeoning field of regenerative medicine.

The outline of the paper is as follows. I begin by briefly placing notions of disease and therapeutic specificity into a medical-historical context. The paper then moves on to propose a model — *the specificity triad* — that draws on Fleck’s concept of “style of thought” and that may allow for a more systematic examination of how such notions guide biomedical reasoning. In the paper’s final section, the specificity triad is used to scrutinize two distinct areas of medicine that have received much scholarly attention in recent years: regenerative medicine and psychiatry. More precisely, I discuss (1) how developmental biology is contributing to the field of regenerative medicine by focusing on the pathophysiology underpinning particular disorders and finding magic bullet *cellular* cures, and (2) how the well-recognized ambiguous epistemic status of psychiatry in contemporary medicine relates to its longstanding inability to do precisely that: to tailor treatments for discrete disorders underpinned by specific pathologies.

### The rise of disease and therapeutic specificity and the advent of biomedicine

Medical historians tell us that the idea of disease and therapeutic specificity is a relatively new arrival in the history of medicine [7]. It is an idea that began to permeate the core of medical reasoning at the turn of the twentieth century, parallel to the unfolding of the sciences of bacteriology and immunology, but it soon became an organizing principle of medical thinking, epitomized, as we have seen, in Ehrlich’s call for “magic bullet” therapeutics. From the vantage point of today, such notions may seem obvious, but for doctors working just a few centuries back this was definitely not the case. In fact, during larger parts of the nineteenth century, doctors were confined to using general treatments, such as bleeding, in combination with an array of drugs, herbs, and poisons whose effects were judged on the basis of their ability to induce visible bodily reactions such as diarrhea, vomiting, perspiration, and urination. The therapeutic value of such treatments was understood and rationalized in the intellectual framework of early-to-mid nineteenth century Western medicine, a framework shared by doctors and patients alike [8]. The underlying rationale was that by provoking potent somatic manifestations the body would regain its normal equilibrium and the patient would be relieved of disease. A classic example is mercury, a popular early nineteenth-century remedy. At low doses it produces obvious bodily reactions such as diarrhea; at high doses or after prolonged treatment, serious mercury intoxication follows, which is associated with copious and uncontrolled production of saliva. Instructively, it was precisely this capacity of mercury to elicit, in a concentration-dependent manner, manifest somatic reactions that by early nineteenth century medicine were hailed as proof of its curative effect involving a mechanism that restored bodily imbalances to a state of healthy equilibrium [8].

But a few decades later, by the second half of the nineteenth century, the framework of Western medicine was undergoing radical changes as bacteriology and clinical medicine were able to link more and more disorders to specific pathogens. Two inaugural discoveries of the “era of specificity” in medicine, attributed to Robert Koch, were the identification of *Bacillus anthracis* (1876) and *Mycobacterium tuberculosis* (1882) as the etiological agents of anthrax and tuberculosis, respectively. Koch famously went on to define a set of methodological rules (known as Koch’s four postulates) for the detection of specific etiological agents of various infectious diseases, which arguably helped to establish specificity as an epistemic space in bacteriology and clinical medicine in the sense that, henceforth, the idea that every infectious disease is a well-defined entity caused by a specific microorganism gained wide acceptance in specialist circles [9].

Around this time, researchers also began to recognize that specific microbial agents elicited not only distinct diseases, but also distinct protective host responses [10]. This insight paved the way for the newly emerging discipline of immunology, which revolved around the investigation of antibodies produced by animals in response to microbial infection [11]. Historians have documented how the establishment of immunology as an independent medical discipline in the late nineteenth century was tightly bound to the idea of how specificity could be applied to objects distinct from — albeit closely linked with — those of bacteriology (microorganisms) and clinical medicine (diseases). Keating and Cambrosio [12] write:

“For immunology to be constituted as an autonomous disciplinary form, it had to be distinguished from both bacteriology and clinical medicine without the foundations or the purpose of either. To remain within a network spanning bacteriology and clinical medicine, it was necessary to speak of species (both species of disease and species of organisms), *and it was necessary to speak of specific antibodies to specific microorganisms that gave rise to specific diseases.*” (p. 320; emphasis added)

Importantly, toward the turn of the twentieth century, specificity in diagnosis and etiology/host response was supplemented with specificity in therapeutics, following the introduction of some disease-specific pharmaceuticals and preventive medical technologies. The most radical therapeutic innovations of this period comprise various vaccines, such as the anthrax and smallpox vaccines introduced in 1883, the rabies vaccine introduced in 1885, and the diphtheria antitoxin introduced in 1890 [13]. Notably, it was by explicit analogy to the antitoxins that Ehrlich, in the first decade of the new century, arrived at his powerful concept of magic bullet drugs: that is, chemically manufactured pharmaceuticals that would seek out the invading enemy without damaging the host [5]. According to Ehrlich, the prospects for radical developments in pharmacology lay wide open: the mission of pharmacologists was “*to learn to aim, aim in a chemical way,*” an idea that has provided momentum to the field ever since.

Following this short overview, it seems fair to conclude that by the early twentieth century bacteriology, immunology, and pharmacology could emerge as (what we today term as) biomedical disciplines by applying notions of specificity to distinct yet connected domains of biological and biochemical inquiry relevant to medicine [12]. But of more relevance to the present argument is – as historian Charles Rosenberg has shown [14] – how the emerging biomedical sciences collectively carried the promise of supplying medicine with a new framework rooted in notions of disease and therapeutic specificity through which to delineate and target the intricate somatic mechanisms of disease.

### Approaching notions of specificity in biomedicine

So how may one analytically approach notions of specificity that appear to play such a pivotal role in the history of biomedical thought? One possibility would be to describe biomedicine’s commitment to specificity as an *ethos*, i.e. as a spirit that guides research in this field. In this way, the “ethos of specificity” would dominate the disciplinary search for discrete particulars (a search of courses not unique to biomedicine). A not altogether dissimilar choice, informed by the work of historians Lorraine Daston and Peter Galison [15], would be to consider specificity an *epistemic virtue* for biomedicine. Daston and Galison use the term epistemic virtue to refer to ideas of what is considered good research in an intellectual and moral sense. Hence the “epistemic virtue of specificity” would denote the value that the collective of biomedical researchers ascribe to specificity (essentially that specificity is a good thing, whereas non-specificity is generally bad).

However, while these approaches may provide valuable insights, describing biomedicine’s commitment to specificity as either an ethos or an epistemic virtue would by itself appear unsatisfactory if one, as is the case here, were interested in delineating in greater detail *how* a commitment to specificity guides biomedical reasoning. Moreover, these approaches fall short in providing an analytic frame for investigating the intricate process through which *links* between specific disorders, mechanisms, and therapeutics are being worked out in biomedicine. That is, if one restricts oneself to an investigation of the “ethos” or “epistemic virtue” of specificity, one arguably runs the risk of missing the central point, which is that not only do discrete entities matter in biomedicine, but also—and perhaps more so—the multiple lines that can be drawn between them.

Another and related option would be to think of specificity in terms of a “regulative principle”; i.e., a common denominator that allows coordination of research efforts across a wide array of bio-disciplines [11]. As we have seen, some historical evidence supports this view, especially in relation to the emergence of immunology as a distinct discipline. In this case, identification of specific host responses contributed to the emergence of immunology as a separate discipline, connected to other biomedical disciplines that share a profound dedication to specificity. But while this approach may provide precious insights regarding academic specialization, it is less useful for investigating how notions of specificity structure the *style of thought* shared by biomedical disciplines – a matter to which the remainder of this paper is dedicated.

### Notions of disease and therapeutic specificity in the style of thought of biomedicine

The concept of style of thought is credited to Ludwik Fleck (1896–1961), a Polish-born physician^a^, bacteriologist, immunologist, and often-regarded pioneer philosopher of science [6]. For Fleck, a style of thought (*Denkstil*) represented the system of beliefs, judgments, methods, emotions, values, and feelings, as well as common problems of interest to a certain community of persons mutually exchanging ideas, or, as he would have it, of a particular thought collective (*Denkkolektiv*). In short, it was “*the entirety of intellectual preparedness for one particular way of seeing or acting, but not for another*[6] (p. 64).” Thus the style of thought is what makes certain ideas thinkable and renders others unthinkable for a certain collective of persons, such as a community of scientists.

A central element in Fleck’s philosophy is that every individual belongs to several thought collectives at once: scientists are not only members of a scientific thought collective; they may participate in political, religious, national, and cultural thought collectives too. Significantly, it is from the multiple interactions within and between thought collectives that the complex matrix of our societies emerges. Moreover, Fleck explains, within any given thought collective it is possible to distinguish between an inner esoteric circle of experts and an outer exoteric circle of non-specialists who gravitate around a certain style of thought. In the case of biomedicine, the inner esoteric circle may be construed as encompassing all scientists and related experts who actively share the style of thought of this science. Typically, such specialists engage in research; they publish and read articles in specialized journals; they attend meetings and conferences; they share methods and reagents; they organize themselves into professional organizations, etc. But we then have the exoteric circle of laymen for which Fleck sets no clear criteria for inclusion, except that members need to gravitate around the thought style and, in some way or another, interact with the esoteric circle of experts.

Strikingly, the contention advanced here, that notions of disease and therapeutic specificity constitute an organizing principle in the style of thought of biomedicine, may actually not be far from Fleck’s own views on the subject, as they are expressed in his once-neglected monograph *Entstehung und Entwicklung einer wissenschaftlichen Tatsache*. This work was first published in German in 1935, but was not translated into English until 1979, following its rediscovery by historians and philosophers of science [6]. The historical case study expounded by Fleck in this monograph revolves around the “serological thought collective” which, Fleck explains, developed the Wassermann reaction, an antibody-based test, into a practical diagnostic test for the detection of syphilis at the beginning of the twentieth century. In his historical investigation of how this test became reified, Fleck uncovers a collection of style elements that he finds to be characteristic of this alleged “serological thought collective,” and that form the conceptual basis for the Wasserman reaction. Among the style elements singled out by Fleck, we encounter: (1) that the reaction between antigen and antibody is specific; (2) the existence of specific disease entities that can be identified by means of systematic clinical and laboratory observation; (3) that every infectious disease is caused by a specific infectious organism; and (4) that host and microorganisms are in conflict. In stark contrast to the views of the “serological thought community” depicted by Fleck, he himself advanced the minority position that infectious diseases should be considered the outcome of dynamic interactions between host and microbe, rather than as specific entities caused by specific bacterial species.

Now, in a reevaluation of Fleck’s work, van den Belt and Gremmen [11] reached the conclusion that the array of style elements identified by Fleck can in principle be reduced to one common denominator: *specificity*. In agreement with my own reading, the authors suggest that “*Fleck’s serological thought style is best seen as part of a more general thought style*” (p.464) that gravitates around the notion of specificity and which, they argue, was shared by clinical medicine, serology, and bacteriology alike. “*Specificity in this broad sense,*” they conclude, “*might be an exemplary expression of what Fleck called a ‘stylistic bond’ existing ‘between many, if not all, concepts of a period*’.” (p.468)

In the literature, Fleck is often mentioned as the first writer who made a compelling case for the sociology of scientific knowledge [16]. In this sense, his ideas are most often compared to those of Kuhn, who launched his paradigm theory almost 30 years later [17] — and indeed Kuhn was influenced by Fleck, which formed the basis for the rediscovery of Fleck in the mid-1960s. But while Fleck and Kuhn definitely share many commonalities, especially concerning the role that both authors ascribe to the social conditioning of thought for scientific fact-making, there are also some significant differences. Perhaps most importantly, when Kuhn discusses meaning-shifts in science, he does this in relation to scientific revolutions that bring about paradigm shifts; that is, a *gestalt* change in which a new interpretation of the body of existing facts completely replaces an existing one, with which it is incommensurable. Fleck, by contrast, envisions styles as much more flexible entities: *as networks of concepts and facts* that may evolve significantly over time [18]. Thus, whereas Kuhnian paradigms are incapable of accommodating anomalies *ad infinitum*, Fleckian styles might in principle evolve beyond recognition when repeatedly challenged.

Fleck’s distinction between *active* and *passive* linkages within a style of thought may be of some help to understand how Fleck envisions styles might evolve over time. Active linkages are the background elements — an ideological prism perhaps — that structure the cognitive acts of individuals of a thought collective. Passive linkages originate when individuals observe the outside world through the ideological prism instilled in them by the thought collective. Fleck also notes how some facts (passive linkages), once they have become certain and reproducible, can mature into active linkages as the thought style evolves. The history of biology and medicine provides numerous examples of this. Think only of how various scientific facts that at an earlier stage seemed uncertain — the nature and structure of DNA, neurochemical transmission in the nervous system, the reprogramming of somatic cells into stem cells — now have come to enable and structure scientific reasoning in entire areas of science.

The main point here is that styles can evolve by turning passive linkages into active ones in a dialectic process involving, on the one side, ideological elements intrinsic to the thought style and, on the other side, the outside material world extrinsic to it. This interpretation of Fleck’s work incorporates both “materialistic” and “idealistic” aspects of his thinking that some commentators have found discordant [18], since it assumes that the bulk of scientific knowledge of a given thought collective is shaped both by constraints imposed by a mind-independent world and by the mind-dependent collective thought style^b^.

### The specificity triad: modeling biomedical reasoning

Following van den Belt and Gremmen’s reading of Fleck [11], the idea of specificity may be viewed as a *stylistic bond* that exists between facts and concepts employed by a biomedical thought collective. But what is the nature of such stylistic bonds, and how can they guide biomedical reasoning?

**Figure 1:**
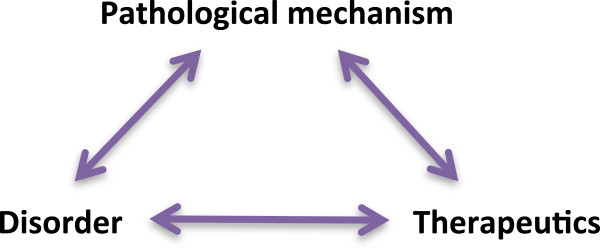
**The specificity triad: a model of a framework in the style of thought of biomedicine.** Disorders, therapeutics, and pathological mechanisms are joined together by links that can be material, theoretical, and semiotic in nature. See text for details.

In this section I propose a model, *the specificity triad*, in an attempt to approach these questions. In brief, the specificity triad refers to a framework in the style of thought of biomedicine created by stylistic bonds, or links, between therapeutics, disorders and pathological mechanisms that, I shall argue, underpin biomedical reasoning (Figure 1). Importantly, while the framework is made up of such links, it is the framework that enables the linking process in the first place. In other words, the framework allows for the establishment of novel links between therapeutics, disorders, and pathological mechanisms. The resultant linkages can then be assimilated into the triad, typically resulting in a further fortification of the framework. Using Fleck’s terminology, the specificity triad can thus be said to model a set of active linkages in the thought style of biomedicine. These active linkages structure thinking, perception, and reasoning by providing a framework for interpreting and manipulating the outside world as it presents itself in the form of drugs, patients, experimental results, etc. (passive linkages), which can then in turn, over time, reify into active linkages, thereby allowing the thought style to evolve.

But what is the nature of these links? What are they made of? I suggest that one may readily differentiate between three types of links: material, theoretical, and semiotic. By material links, I refer to connections made from material entities. These would include such things as drugs, molecules, germs, experimental equipment, and diagnostic tests that all act to join disorder, pathological mechanism, and therapeutics together in the knowledge system of biomedicine. For example, after Alexander Fleming made his now legendary observation in 1928 that the *Penicillium* mold obliterates the staphylococcus bacterium responsible for a great many infectious deaths, a firm connection was established between a specific therapy, a discrete disorder, and an underlying pathological mechanism. Similarly, the discovery of insulin, its subsequent synthesis and its employment as a treatment for diabetes contributed to the creation of a framework within which specific connections between therapeutics, disorder, and pathologic mechanism could be further elaborated in endocrinology and later in regenerative medicine (see below). It is easy to see how such material connections contribute to the reinforcement and evolution of a framework that allows researchers to further pursue issues related to, in these cases, infectious diseases and diabetes. However, it would also seem clear that the fabrication of material links in the first place follows a script revolving around notions of specificity.

By theoretical links I refer to connections made via explicit mechanistic models, including various disease and treatment models. Such models usually act to connect therapy, somatic mechanism and disorder on a *conceptual* level. Mechanistic models are often based on prior established material links. For example, in the case of depression, researchers have used the biochemical and therapeutic effects of antidepressant drugs to devise various pathophysiological theories about the disorder [19]. But theoretical links can also generate material links; for example when pathophysiological theories provide impetus to drug development. Significantly, theoretical links contribute to reinforcement and evolution of the framework which enables scientists to collectively engage in research.

Semiotic, finally, refers to connections through language. For instance, the very act of calling a drug an *anti-*depressant automatically connects depressive disorder to this particular type of therapeutics [20]. Similarly, commonplace gene-talk about “genes for” certain disorders, such as dyslexia or diabetes connects, by means of rhetoric, pathological mechanism to disorder [21]. Or, to take another example from psychiatry, talk about “chemical imbalances” in relation to mood disorders connects pathological mechanism and disorder without much need for an explicit scientific theory [22] (see below). As with material and theoretical links, I suggest that semiotic links are contingent on the existence of an *a priori* framework that makes their fabrication possible and acceptable.

A possible counterargument is that some—perhaps even all—links may at once be construed as material, theoretical, and semiotic in nature. For instance, one could ask if not all material links are theory-laden and language-dependent? Should this indeed be the case, it would perhaps be better to speak of material-theoretical-semiotic links, or mechanistic links or simply links, rather than separating them into seemingly neat classes. However, as philosopher Ian Hacking [23] has argued, the common truism that all observations in science are theory-laden needs to be better specified. While it is probably true that our ability to interpret and describe the world depends on the state of our knowledge — that what we “see” is contingent upon what we “know” — our state of knowledge should not be confused with our explicit theories about the world. Thus we may learn to “see” things without explicit scientific theories to accommodate those observations. Similarly, to claim that material links are language-dependent is not the same as saying that material links are determined by language, or that they can be reduced to language [21]. Consequently, condensing material, theoretical and semiotic links into a single category may not be particularly useful, but instead contribute to concealing important distinctions between, say, the effect of drugs on molecules or symptoms (material), the way those effects are explicitly incorporated into mechanistic models (theoretical), and the metaphors and rhetoric sometimes used when speaking about those effects (semiotic).

### Regenerating development: the specificity triad in regenerative medicine

Arguably, a main virtue of the specificity triad model is how it focuses the analytic gaze on the various material, theoretical, and semiotic links forged between therapeutics, disorder, and pathophysiology. In the following sections of this paper the specificity triad is used to scrutinize two distinct areas of medicine: regenerative medicine and psychiatry. In this first part I discuss how such links are currently worked out at the intersection of developmental biology and the burgeoning field of regenerative medicine, which is concerned with replacing or regenerating human cells, tissues, or organs, to restore or establish normal function. The point is to show how this intermingling is transforming developmental biology into a core biomedical discipline. Paraphrasing Ehrlich, the prospect of developmental biology appears, in this view, to lay wide open: the mission is to learn to aim, aim in a *cellular* way, an idea that is now providing strong momentum to the field.

Much has been written about the history of developmental biology, or embryology as the field was known until the 1950’s, and its complex relation to other fields (e.g. [24,25]). Against this background it seems fair to conclude that over the preceding century the bulk of work in developmental biology has had little relation to biomedical concerns: the general focus was not on finding cures for specific human disorders or delineating specific pathologic mechanisms, but rather on uncovering general principles of organism development that could explain the reproducibility of development within species. That said, there have undoubtedly been important areas of overlap between developmental biology and biomedical efforts, for example in research on teratology [26]. Significantly, this area of overlap has increased over the last decades following the realignment of genetics and developmental biology [27], epitomized in the 1995 and 2002 Nobel Prizes awarded to developmental biologists for research on genetic control of embryonic development [28,29]. Crucially, as argued below, this intermingling has since deepened further, to the extent that leading developmental biologists are now calling on their peers to truly contribute to explaining disease and treating it by finding magic bullet *cellular* cures for specific disorders.

Thus in the opening pages of the first 2013 issue of the journal *Development* – launched in 1953 as the *Journal of Embryology and Experimental Morphology,* and ever since a leading journal in the field of developmental biology – Editor-in-Chief Oliver Pourquié stressed the great opportunities that lay ahead for developmental biologists to contribute to medical treatments and the understanding of human pathologies [30]. He appropriately called his piece *Regenerating development*, which alluded both to the journal *Development’s* alleged need to re-embrace the growing field of stem cell biology – which he described as an offshoot of developmental biology – *and* to the prospect for developmental biology as a discipline to make significant contributions to the field of regenerative medicine. He did this against the backdrop of developmental biologist John Gurdon and stem cell biologist Shinya Yamanaka having shared the 2012 Nobel Prize for their work on stem cells and reprogramming. This work spanned more the four decades. In 1962, incidentally the same year Yamanaka was born, Gurdon reported in the pages of the *Journal of Embryology and Experimental Morphology* that he could reprogram the nucleus of an adult frog cell to a totipotent embryonic state by nuclear transplantation [31]. This set the stage for subsequent research on reprogramming of cells, including from Yamanaka’s group, which in 2006 succeeded in identifying a small number of genes within the genome of mice that seemed decisive in this process [32]. As noted by Pourquié, this line of work revolutionized the field of developmental biology conceptually because it “*shows that it is possible to reverse the course of development and differentiation*” (p.1). However, it also revolutionized the field in the sense of aligning it with biomedical efforts: combined with recent developments in organ culture from stem cells *in vitro*, reprograming of cells “*means that recreating human organs* in vitro *is a real and achievable goal*” (p.1). Pourquié writes:

“*This should open a new era in which the uncharted territory of human developmental biology will be explored. In addition, it will allow us to produce differentiated cells of all human lineages at all stages of differentiation, raising the possibility of establishing* in vitro *models of human diseases to study their pathophysiology and to screen for new treatments or cures. Finally, these advances should favour the development of cell therapy and regenerative medicine, potentially allowing the replacement of missing cells of body parts with cells from organs engineered* in vitro*. These are some of the major challenges that are now within reach thanks to Gurdon and Yamanaka’s discoveries*” (p.1).

In sum, in this view, the prospect of developmental biology as a biomedical enterprise lies in (1) providing models of human pathologies and in testing and developing new therapies for these disorders, and (2) replacing missing cells of body parts with cultured cells. Crucially for my argument, the underlying idea is that recent breakthroughs have created a niche for developmental biologists where they can fortify links between disorder, pathophysiology, and therapeutics for a wide range of human diseases and hence contribute to the overarching biomedical enterprise.

Indeed, such efforts are already underway. A recent review published in the prestigious journal *Cell Metabolism* summarizes these efforts as follows in regard to research on various subgroups of diabetes [33]:

*“The landmark discovery of induced pluripotent stem cells (iPSCs) by Shinya Yamanaka has transformed regenerative biology. Previously, insights into the pathogenesis of chronic human diseases have been hindered by the inaccessibility of patient samples. However, scientists are now able to convert patient fibroblasts into iPSCs and differentiate them into disease-relevant cell types. This ability opens new avenues for investigating disease pathogenesis and designing novel treatments. In this review, we highlight the uses of human iPSCs to uncover the underlying causes and pathological consequences of diabetes and metabolic syndromes, multifactorial diseases whose etiologies have been difficult to unravel using traditional methodologies”* (p.775).

The idea, then, is that scientists have begun to use stem cells – in this case induced pluripotent stem cells (iPSC) – to establish firm connections between various disorders, pathologies and treatments. The text goes on to specify the numerous *material* links that are being established, in particular by reprogramming somatic cells from patients with various forms of diabetes into so-called diabetes-induced pluripotent stem cells (DiPSCs) which can then be used to identify “*new genes and pathways that contribute to diabetes pathogenesis in humans and novel drugs that target these pathways*” (p.780). However, this work can also be seen as elaborating *theoretical* links between the triad’s nodes. Thus, conceptually, the text advances the notion of DiPSCs as a novel and more attractive disease model in which to screen for therapeutics compared with conventional rodent models, which are chided for not replicating the complexity of human pathogenesis. Moreover, echoing Pourquié, the review crafts *semiotic* links by means of the rhetorical act of making future-orientated claims about regenerative medicine, stating for example that DiPSCs “*may one day provide a source of patient-specific replacement cells for β-cells lost to diabetes…*” (p.778). This latter point is vital because such hopes are currently informing a major collective biomedical research effort concerned with how these cells can be mobilized to permanently regenerate damaged tissue without injuring the human recipient. In conclusion, we can see how the intermingling of developmental biology and medicine is contingent on a framework of specificity, but also how developmental biology carries the promise of invigorating biomedicine by establishing fresh links between disorders, therapeutics, and pathologies. The next section explores what may happen in case such promises remain unfulfilled.

### When things fall apart: the specificity triad in psychiatry

Psychiatry is the branch of medicine concerned with the diagnosis, treatment, and prevention of mental and emotional disorders. Compared with other medical specialties, psychiatry was relatively late in absorbing notions of disease and therapeutic specificity into clinical practice and research. Historians and psychiatrists have noted how this strongly contributed to the ambiguous epistemic status of this medical specialty in the first half of the twentieth century [4,34]. Yet social scientists have argued that the same can be said about the epistemic status of psychiatry in contemporary medicine [35,36]. The present analysis will discuss how this ambiguity relates to psychiatry’s long-standing struggle to establish firm connections between specific disorders, therapeutics, and pathologies.

Historians tell us that the first psychiatric treatment to be generally recognized as specific for a distinct psychiatric disorder was introduced as early as 1917 by Viennese psychiatrist Julius Wagner-Jauregg [34]. In the course of his experimenting with psychiatric patients, Wagner-Jauregg discovered that the fever induced by malaria might cure people suffering from General Paralysis of the Insane, or Dementia Paralytica, a lethal psychiatric disorder caused by syphilis infection in the brain, which, as it turned out, could be attenuated by elevating body temperature, a discovery that gained Wagner-Jauregg the Nobel Prize ten years later in 1927. The importance of the discovery of “fever cure” for psychiatry should not be underestimated. For the first time, psychiatrists had proof of principle that psychiatric disorders and treatments were not necessarily dissimilar from those of other medical specialties. As in the rest of medicine, it therefore also seemed reasonable for psychiatry to engage in a search for specific treatments for specific disorders with specific causes [34]. This motivation was strengthened a few decades later when insulin coma, shock therapies, and later penicillin were introduced into psychiatric practice [4]. By 1940, it had become widely assumed in specialist circles that hour-long insulin comas provoked by injections of the hormone insulin constituted a specific and efficient treatment for schizophrenia. Simultaneously, shock treatments, first chemically and then electrically induced (so-called ECT), were considered by many experts to be specific and effective for severe depressions. A few years later, near the end of World War II, penicillin supplanted fever cure as specific treatment for neurosyphilis. “*By the mid-twentieth century,*” concludes psychiatrist Joanna Moncrieff in her historical overview of biological treatments in psychiatry during this period, “*psychiatrists finally believed that they could resolve the problems experienced by people under their care by acting on what they presumed was the bodily basis of the problem*” [4] (p.40).

Importantly, for the purpose of this paper, the belief that psychiatric treatments acted on the somatic basis of disorder provided strong impetus for biological research in psychiatry. Moreover, this belief was reinforced after the serendipitous discovery of so-called antidepressant and antipsychotic medications in the 1950s [37]. To cite one important example, such ideas underpinned the 1969 decision by the US National Institute of Mental Health (NIMH) to set up a large program on the “psychobiology of depression” [19]. This program eventually enrolled about 1400 patients with the aim of solving a set of pertinent medical questions related to depression and antidepressants [38]. In the preceding years NIMH staff had become increasingly impressed by the “*burgeoning productivity of biochemists, biological psychiatrists, and psychophysiologists in this field, and the potential these results might have on its [depression’s] understanding and treatment*” [39] (p.x)*.* The NIMH therefore decided to sponsor a research effort that would concentrate on three major objectives: “*(1) nosology — the development of a sound, reliable system of classification of the depressive disorders; (2) genetics — the design of studies to permit the definitive test of hypotheses concerning the role of genetics; and (3) pathophysiology — the investigation of the role of specific biochemical, neurophysiological, and endocrine mechanisms implicated in the etiology of depression*” [40] (p.766). Regarding the objective to investigate pathophysiology, it was felt that “*without such* [pathophysiological] *hypotheses to guide research, new, more effective treatment methods and deeper understanding of the affective disorders probably would not be realized*” [40] (p.767). In sum, the explicit aim was to explore empirically and conceptually the biologic basis of affective disorders; i.e., to establish *material* and *conceptual* links between disorder and pathophysiology, including genetics, as this could offer a firm basis for developing efficient treatment methods.

But despite this extensive research undertaking that lasted for over a decade, the program proved disappointing since researchers failed to find the biological basis of affective disorders [19]. Nonetheless, the undertaking left a long-lasting impression on the field as it helped transform psychiatric nosology [41]. A major issue had been to find a set of operational criteria for psychiatric diagnosis that would permit researchers to reliably assign patients to specific disorder categories (e.g. depression, mania, schizophrenia). It was believed that a reliable diagnostic system needed to be in place before biomedical research in psychiatry could progress significantly. To this end, seminal psychiatrists (e.g. Eli Robins, Robert Spitzer and Jean Endicott) went on to develop the so-called Research Diagnostic Criteria, a collection of operational criteria for psychiatric diagnosis used by the researchers in the program [42,43]. Crucially, the Research Diagnostic Criteria and the intra-institutional and individual ties established during its development, notably between Robert Spitzer in New York, Eli Robins and Sam Guze in St. Louis, and Gerald Klerman in Boston, laid the foundation for the work that culminated in the *DSM III*, *The Diagnostic and Statistical Manual of Mental Disorders*, which was published 5 years later, in 1980 [44-46]. As noted by commentators from a wide spectrum of academic positions, including biological psychiatry, publication of the DSM III brought with it a reconfiguration of world psychiatry into a “justifiable” medical specialty that, like the rest of medicine, could be represented as treating legitimate disorders, which, according to the norms of biomedicine, should have discrete boundaries and be linked to specific underlying causes [45,47,48]. Nancy Andreasen, prominent biomedical psychiatrist with close ties to the *DSM III* task force responsible for drafting the manual, made this view explicit when she, a few years after the launch of the *DSM III*, outlined the creed of specificity as applied to psychiatry by crafting *theoretical* and *semiotic* links between psychiatric disorders, therapies, and pathologies:

“The major psychiatric illnesses are diseases…caused principally by biological factors, and most of those factors reside in the brain…As a scientific discipline, psychiatry seeks to identify the biological factors that cause mental illness…*This model assumes that each different type of illness has a different specific cause…Because these diseases are considered to be of biological origin, the therapy is seen as correcting an underlying biological imbalance*” [49] (p.29-30; italics in original).

By the early 1980s, leading professionals and researchers had thus collectively rejected notions of non-specificity, replacing them with a seemingly unanimous discourse on disease and therapeutic specificity, perhaps nowhere more succinctly formulated than in the above quote from Andreasen. However, despite assurances from leading psychiatrists, researchers continued to experience difficulties in establishing firm *material* connections between disorders, therapies, and pathologic mechanisms. This was, for example, made explicit in a 2002 consensus statement by a workgroup charged by the NIMH with analyzing major gaps in the knowledge of mood disorders and their treatments, and with formulating a series of recommendations for the NIMH to help overcome obstacles [50]. The workgroup described the limited progress in the field since the serendipitous discovery of effective psychotropic medications in the 1950s as follows (p.503):

“We still do not understand with certainty how those medications produce their desired clinical effects. We have not introduced newer medications with fundamentally different mechanisms of action than the older agents. We have not identified the genetic and neurobiological mechanisms underlying depression and mania, nor do we understand the mechanisms by which nongenetic factors influence these disorders. We have only a rudimentary understanding of the circuits in the brain responsible for the normal regulation of mood and affect, and of those circuits that function abnormally in mood disorders.”

The situation ten years later appears almost equally disappointing, although there has been some recent progress, especially regarding the putative role of various brain circuits in some psychiatric states [51]. Thus biomarkers are still lacking for the main psychiatric diagnoses [52], and most major drug companies have dropped psychotropic research, partly due to the lack of viable biological hypotheses [53]. From the perspective of neuroscience, a main obstacle to progress is claimed to be the absence of an objective diagnostic and classification system in psychiatry. It is argued that, unlike the rest of medicine that typically identifies and categorizes diseases on the basis of physiological and histological abnormalities, psychiatry identifies its disorders with subjective behavioral tests that have not been rigorously validated. This results in heterogeneous patient cohorts with which it is notoriously difficult to work. Neuroscientist Husseini Manji has argued: *“The heterogeneity implicit in the current classification schemes is a probable reason for the limited success of clinical studies, at the levels of treatment, neurobiology and genetics*” [54] (p.189). Similarly, other prominent researchers have pointed out how the current, non-biological diagnostic system “*poses obvious obstacles to the interpretation of genome-wide association studies, as well as neuroimaging and post mortem investigation*” [55] (p.894) The perhaps most broadcasted statement of this sort came from Thomas Insel [56], NIMH director, who prior to the imminent launch of the *DSM V* wrote on his NIMH blog that the manual is “*at best, a dictionary, creating a set of labels and defining each“* and that *”its weakness is its lack of validity*.” He said that the NIMH would reorient its research away from the manual because “*unlike our definitions of ischemic heart disease, lymphoma, or AIDS, the DSM diagnoses are based on a consensus about clusters of clinical symptoms, not any objective laboratory measure.*”

Anthropologist Andrew Lakoff has argued that the failure of neuroscience research to provide biological validation for psychiatry’s diagnostic categories poses not only a challenge to the legitimacy of psychiatric knowledge, but questions the very existence of its objects [35]. He quotes a leading psychiatric expert who expressed “*frustration at the epistemological status of psychiatry*” and who complained that: “*(…) In no other branch of medicine have investigators (and practitioners) been called on to demonstrate time and time again that the diseases they study really are diseases.*” Ostensibly, such statements expose the ambiguous epistemic status of psychiatry in contemporary medicine. This ambiguity, I contend, is testimony to the incongruity between, on the one hand, the style of thought of biomedicine that underpins biological research in psychiatry and, on the other hand, the long-standing nebulous nature of the links between specific disorders, pathologies, and therapeutics in psychiatry.

## Conclusion

This article has developed a frame that may allow for a fruitful analysis of how notions of disease and therapeutic specificity guide biomedical reasoning. To that end, I drew on the work of historians of medicine who have suggested that ideas of tailored treatments for discrete disorders with specific causes began to permeate the core of medical reasoning at the turn of the twentieth century. However, to further theorize the role of such ideas in guiding biomedical reasoning I turned to the philosophy of science and, more precisely, to Ludwik Fleck’s ideas about styles of thought. Based on Fleck’s distinction between passive and active linkages within thought styles, I proposed the specificity triad as a model of a framework that guides biomedical reasoning. More precisely, the specificity triad refers to a framework in the style of thought of biomedicine created by links of various kinds (material, theoretical, and semiotic) between therapeutics, disorders, and pathological mechanisms. It was suggested that this framework provides a general scaffold for interpreting and manipulating the outside world as it presents itself in the form of, e.g., drugs, patients, and experimental results. Such passive linkages, in Fleckian terms, can then reify over time into active linkages, thereby allowing the style of thought of biomedicine to evolve.

Fleck clearly believed that thought styles existed “out there” and that they, as such, conditioned the cognition of working scientists. Fleck’s position has subsequently been challenged. For example, Jonathan Harwood has argued that thought styles are best understood as analysts’ constructs, and as such they cannot condition anybody’s thinking except our own [18]. In this view, styles are said to differ from theories, concepts, or techniques that are readily recognized by working scientists as a guide to their everyday work. Some have nevertheless maintained that the concept of style of thought may still be of significant heuristic value insofar as it helps simplify the complexity of the body of knowledge under scrutiny into an amenable format that allows the analyst to focus on some deeply embedded assumptions that underpin reasoning [57,58]. The specificity triad should be understood in this particular fashion, and it is because of that that I have referred to it as a *model*. From this perspective it may be worth reiterating that one of the merits of this model is how it draws attention to the various and more or less fragile material, theoretical, and semiotic links forged by biomedicine between therapeutics, disorders, and pathophysiology. Here I have tried to demonstrate this using the examples of regenerative medicine and psychiatry.

Lastly, it is important to emphasize that this model is by no way presented as a general challenge to the accuracy of the content of the biomedical sciences. On the contrary, I firmly believe that biomedical reasoning and research emerged and endured because of its productivity – as for example seen in the burgeoning field of regenerative medicine – and because it more often than not structures the world in a reliable and predictive way. However, biomedicine has not always succeeded in fulfilling its aspirations, as the case of psychiatry unmistakably shows. The reason and possible solution for this is still a matter of intense debate among psychiatrists, philosophers, historians, neuroscientists, and others. Thus one widely debated proposition for improving the validity of psychiatric diagnosis advanced by social scientists is to explicitly incorporate social values alongside scientific components in the concept of disorder [59]. Another proposition, but from the natural sciences, is to replace the existing framework with a framework likely more relevant to the nature of psychiatric disorders (and many other disorders) based on the existence of diverse but connected causal processes at multiple levels (e.g. molecular, physiological, psychological, social) that link disorders, mechanisms, and therapeutics [60]. Yet, arguably, it is to a large extent this incongruence between the style of thought of biomedicine and the reality of psychiatric research, as addressed here, that makes psychiatry more vulnerable to social and biological criticism than other medical specialties. Thus as Charles Rosenberg has argued, in today’s biomedicine, diseases that fail to be associated with a specific cure or with a specific pathological mechanism that can be approached with seemingly objective laboratory methods or imaging techniques, run the risk of being degraded to a lesser epistemological status [14].

### Endnotes

^a^For contrasting views on the value of Fleck’s biomedical scholarship and ethical conduct, and of its relation to his epistemology, see [61-64].

^b^This reading eschews more radical constructivist claims occasionally made by Fleck. But those claims are incongruent with some weaker versions of constructivism that Fleck advances elsewhere in his texts, and even more so with his own scientific practice, especially his later work [18]. One possible interpretation is that the inconsistencies in Fleck’s writings reflect an unresolved tension between Fleck the philosopher that stresses relativism and idealism and Fleck the scientist that stresses realism and materialism.

## Competing interests

The author declares that he has no competing interests.
